# Association between the systemic inflammation response index and mortality in the asthma population

**DOI:** 10.3389/fmed.2024.1446364

**Published:** 2024-09-03

**Authors:** Feng Xu, Hui Jiang, Fanglan Li, Yan Wen, Pan Jiang, Feng Chen, Yongwen Feng

**Affiliations:** ^1^Department of Intensive Care Unit, Shenzhen Guangming District People's Hospital, Shenzhen, Guangdong, China; ^2^Department of Pulmonary and Critical Care Medicine, Shenzhen Guangming District People's Hospital, Shenzhen, Guangdong, China; ^3^Department of Stomatology, Shenzhen Guangming District People's Hospital, Shenzhen, Guangdong, China

**Keywords:** systemic immune-inflammation index, mortality, asthma, NHANES, association

## Abstract

**Background:**

As a novel indicator of inflammation, the relationship between the systemic immune-inflammation index (SIRI) and mortality in patients with asthma remains uncertain. Our study aimed to explore the association between SIRI and mortality in asthma patients.

**Methods:**

Data from the National Health and Nutrition Examination Survey (NHANES) for US adults from 2001 to 2018 were included in this study. Then, we divided all patients into three groups based on SIRI tertiles and used multivariable weighted Cox regression analysis, smoothing curve fitting, survival curve analysis, and subgroup analysis to investigate the relationship between SIRI and asthma.

**Results:**

A total of 6,156 participants were included in the study, with each SIRI tertile consisting of 2052 individuals. Asthma patients with higher SIRI levels were older, had a higher level of education, were more likely to be married, and had a higher chance of being smokers. In Cox proportional-hazards models, the highest SIRI group showed higher hazard ratios (HRs) for all-cause mortality in individuals with asthma after adjusting for potential confounders. The restricted cubic spline analysis indicated a non-linear relationship between SIRI and all-cause mortality. The Kaplan–Meier survival curves showed that patients with higher SIRI levels had a higher risk of all-cause mortality. Subgroup analyses revealed SIRI’s association with all-cause mortality across various demographics, including age, sex, race, education levels, smoking status, and marital status.

**Conclusion:**

Our study provides evidence for the relationship between SIRI and mortality in asthma patients. SIRI may potentially serve as a predictive tool for evaluating asthma mortality rates.

## 1 Introduction

Asthma, a chronic inflammatory disease of the airways, is characterized by reversible constriction and hyperresponsiveness of the airways. The disease presents a considerable global healthcare burden, affecting an estimated 334 million people worldwide and increasing every year (1). Exacerbations of asthma result in large numbers of emergency department visits, while improved control of the disease reduces healthcare expenses. Therefore, it is important to understand the causes of asthma.

The systemic immune-inflammation index (SIRI), as an inflammation index, is composed of peripheral blood neutrophil, monocyte, and lymphocyte counts (2). SIRI is widely used for the survival and mortality of various diseases as a predictive indicator in many studies. In a prospective study including 298 postoperative colorectal patients, compared to other serum inflammatory biomarkers, SIRI had a higher predictive ability for survival outcomes (3). In a prospective study including 52 patients diagnosed with advanced lung adenocarcinoma, Wang et al. found that the low SIRI level had a prolonged progression-free survival (4). A meta-analysis revealed that high SIRI levels could predict adverse survival outcomes in breast cancer patients (5). Furthermore, SIRI can provide better prognostic discrimination correlating with mortality risk not only in chronic heart failure patients (6) but also in cardiovascular and cerebrovascular disease patients (7–10). In addition, elevated SIRI levels are also associated with the incidence and mortality rates of chronic kidney disease (CKD) (11).

Although there is emerging evidence that the risk of all-cause mortality in asthma depends on the highest quartile of SIRI compared to the lowest quartile of SIRI (12), the research on the relationship between SIRI and mortality risk in asthma has not been evaluated as far as we know. Therefore, to investigate the relationship between SIRI and mortality rates in asthma patients, we use data from the National Health and Nutrition Examination Survey (NHANES) spanning from 2001 to 2018, aiming to find new valuable prognostic indicators.

## 2 Research design and methods

### 2.1 Study population

**Figure 1:**
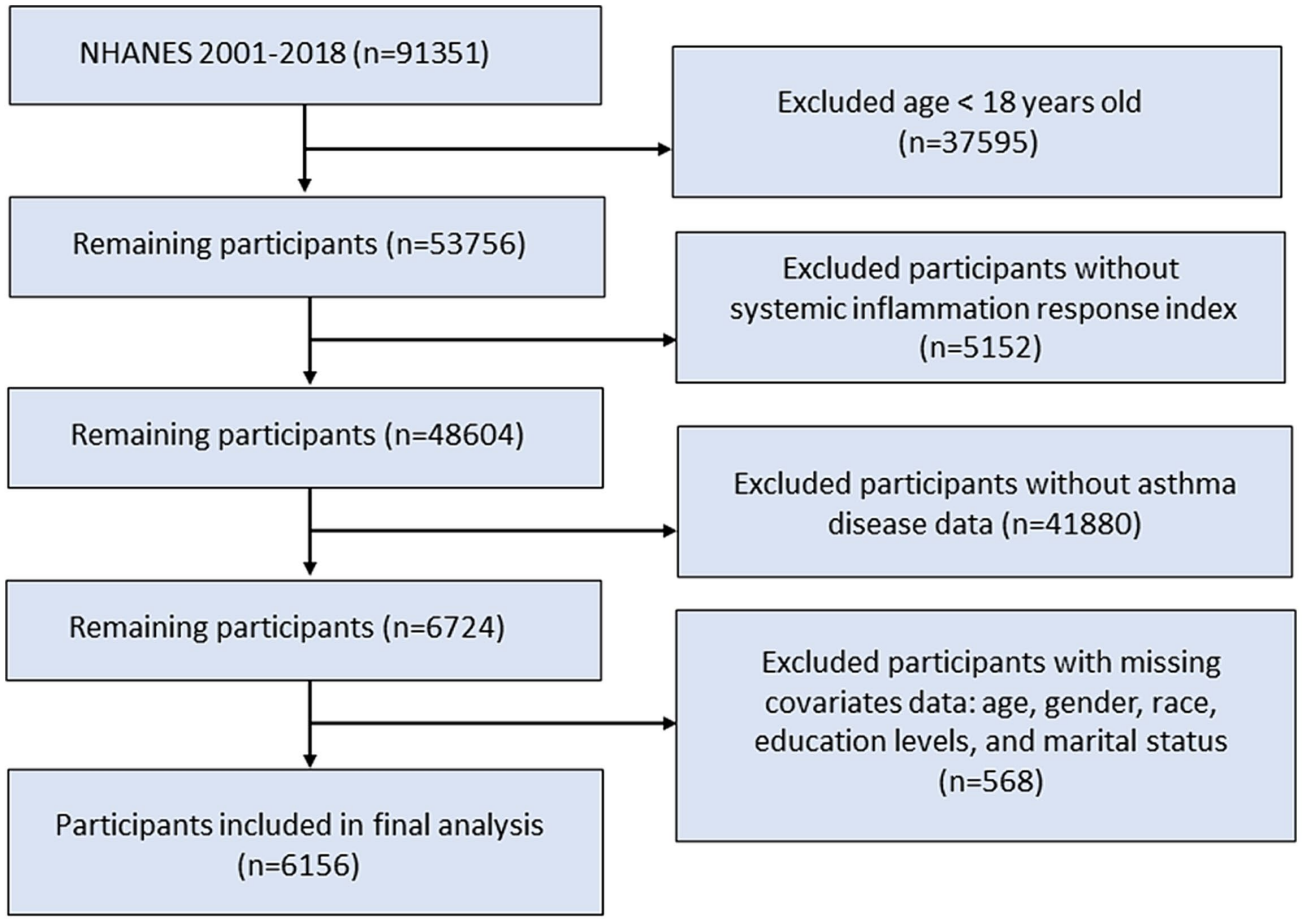
Flowchart of study population selection.

The NHANES provided cross-sectional data to assess the nutritional and health status of the non-institutionalized civilian population of the United States. Our study utilized data from the continuous NHANES survey, spanning 11 survey cycles conducted between 2001 and 2018, with a total of 91,351 participants. A total of 53,756 eligible participants aged 18 years and older were included in the study population. We excluded patients without SIRI or asthma disease data. Additionally, participants without records of age, sex, race, marital status, or education level were excluded from the study. Finally, 6,156 participants were included in this study (Figure 1).

### 2.2 Assessment of mortality

All-cause mortality data were obtained by linking to the National Death Index as of April 26, 2022.

### 2.3 Statistical analysis

SIRI was calculated using the formula (neutrophil count × monocyte count)/lymphocyte count. Participants were equally classified into three groups based on the values of SIRI. The adjusted hazard ratios (HRs) and 95% confidence intervals (95% CIs) were calculated using the Cox proportional-hazards regression model. This observational study used three models based on the Strengthening the Reporting of Observational Studies in Epidemiology (STROBE) guidelines (13). Model I was adjusted for none. Model II was adjusted for age, sex, and race. Model III was adjusted for all variables in Model II and included other risk factors such as marital status, education level, and smoking status. Furthermore, the Kaplan–Meier survival curves for all-cause mortality stratified by the values of the SIRI index were generated. Smooth curve fitting (penalized spline method) examined the non-linearity association between the SIRI index and all-cause mortality. All data analyses were performed using R version 4.2.2, and *p*-values less than 0.05 indicated statistical significance.

## 3 Results

### 3.1 Subject characteristics

The baseline characteristics of the cohort were analyzed based on the changes in SIRI tertiles, as shown in Table 1. This analysis included 6,156 patients, with each tertile based on 2,052 individual observations. Compared to the lowest SIRI tertile group, patients in the highest SIRI tertile group had a higher average age, were more likely to be married, had a higher level of education, and had a higher chance of being smokers. Additionally, the male-to-female ratio was similar in all SIRI groups, but the patients in the highest SIRI tertile group were predominantly non-Hispanic white. Furthermore, all baseline variables showed statistically significant differences between the SIRI groups (*p* < 0.05).

**Table 1:** Demographic and clinical characteristics according to systemic inflammation response index (SIRI).

Variables	SIRI			*P*-value
	Q1	Q2	Q3	
Number of patients	2052	2052	2052	
SIRI	0.58 ± 0.17	1.07 ± 0.15	2.27 ± 1.27	<0.001
Age (%)				<0.001
<60	1,534 (74.76%)	1,441 (70.22%)	1,325 (64.57%)	
≥60	518 (25.24%)	611 (29.78%)	727 (35.43%)	
Sex (%)				<0.001
Male patients	760 (37.04%)	865 (42.15%)	923 (44.98%)	
Female patients	1,292 (62.96%)	1,187 (57.85%)	1,129 (55.02%)	
Race (%)				<0.001
Mexican	185 (9.02%)	229 (11.16%)	198 (9.65%)	
Hispanic	184 (8.97%)	201 (9.80%)	191 (9.31%)	
Non-Hispanic White	731 (35.62%)	1,026 (50.00%)	1,191 (58.04%)	
Non-Hispanic Black	745 (36.31%)	404 (19.69%)	321 (15.64%)	
Others	207 (10.09%)	192 (9.36%)	151 (7.36%)	
Education level (%)				0.015
≤High school	895 (43.62%)	909 (44.30%)	970 (47.27%)	
College	689 (33.58%)	695 (33.87%)	698 (34.02%)	
>College	468 (22.81%)	448 (21.83%)	384 (18.71%)	
Marital status (%)				0.009
Not married	1,570 (76.51%)	1,633 (79.58%)	1,645 (80.17%)	
Married or living with a partner	482 (23.49%)	419 (20.42%)	407 (19.83%)	
Smoking status				
Yes	942 (45.91%)	982 (47.86%)	1,188 (57.89%)	<0.001
No	1,110 (54.09%)	1,070 (52.14%)	864 (42.11%)	

Mean ± standardized differences (SD) for continuous variables, percentages (%) for categorical variables. SIRI, systemic inflammation response index.

### 3.2 Association between the SIRI index and mortality

**Figure 2:**
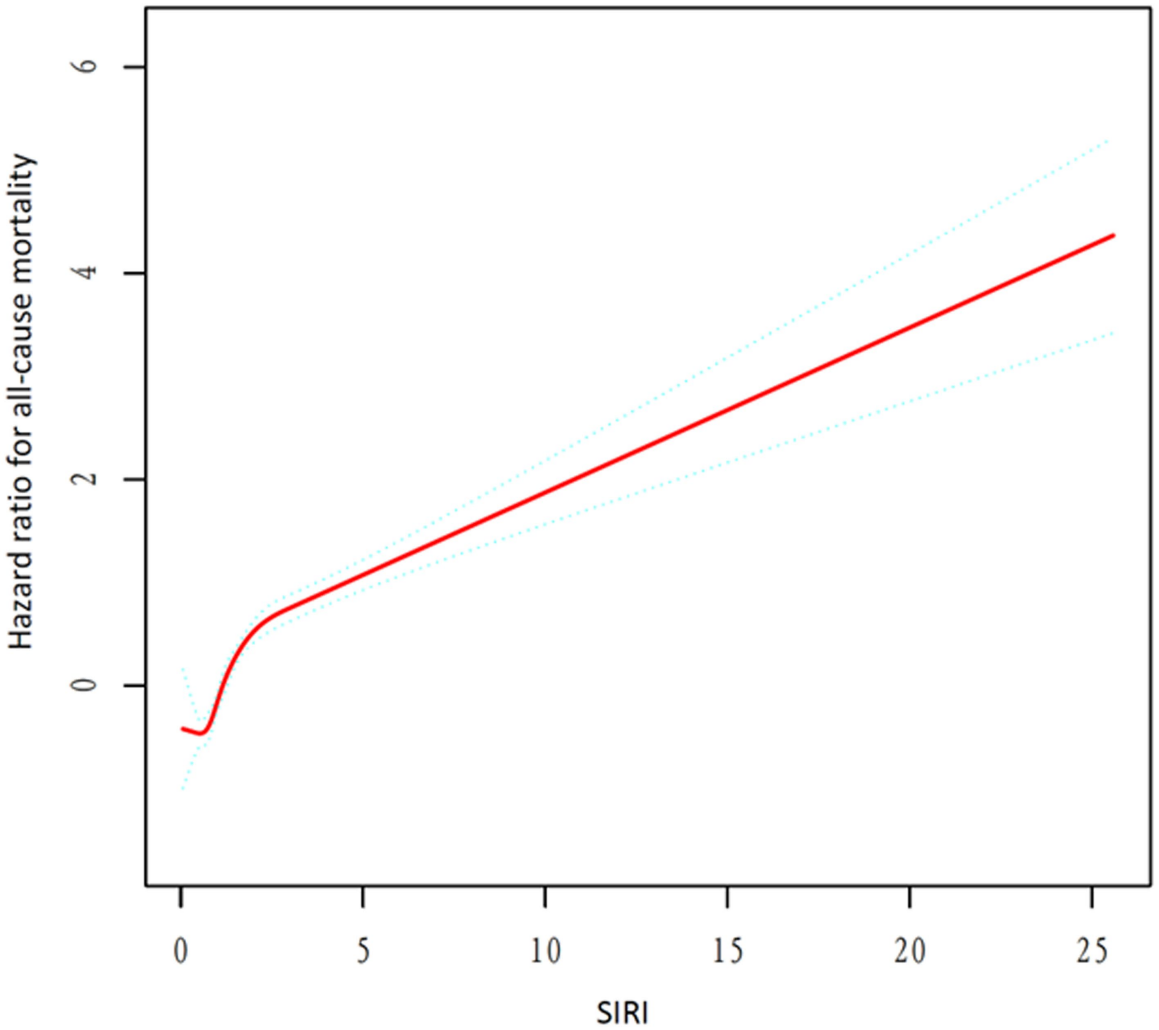
Restricted cubic spline analysis of the association between SIRI and all-cause mortality.

**Figure 3:**
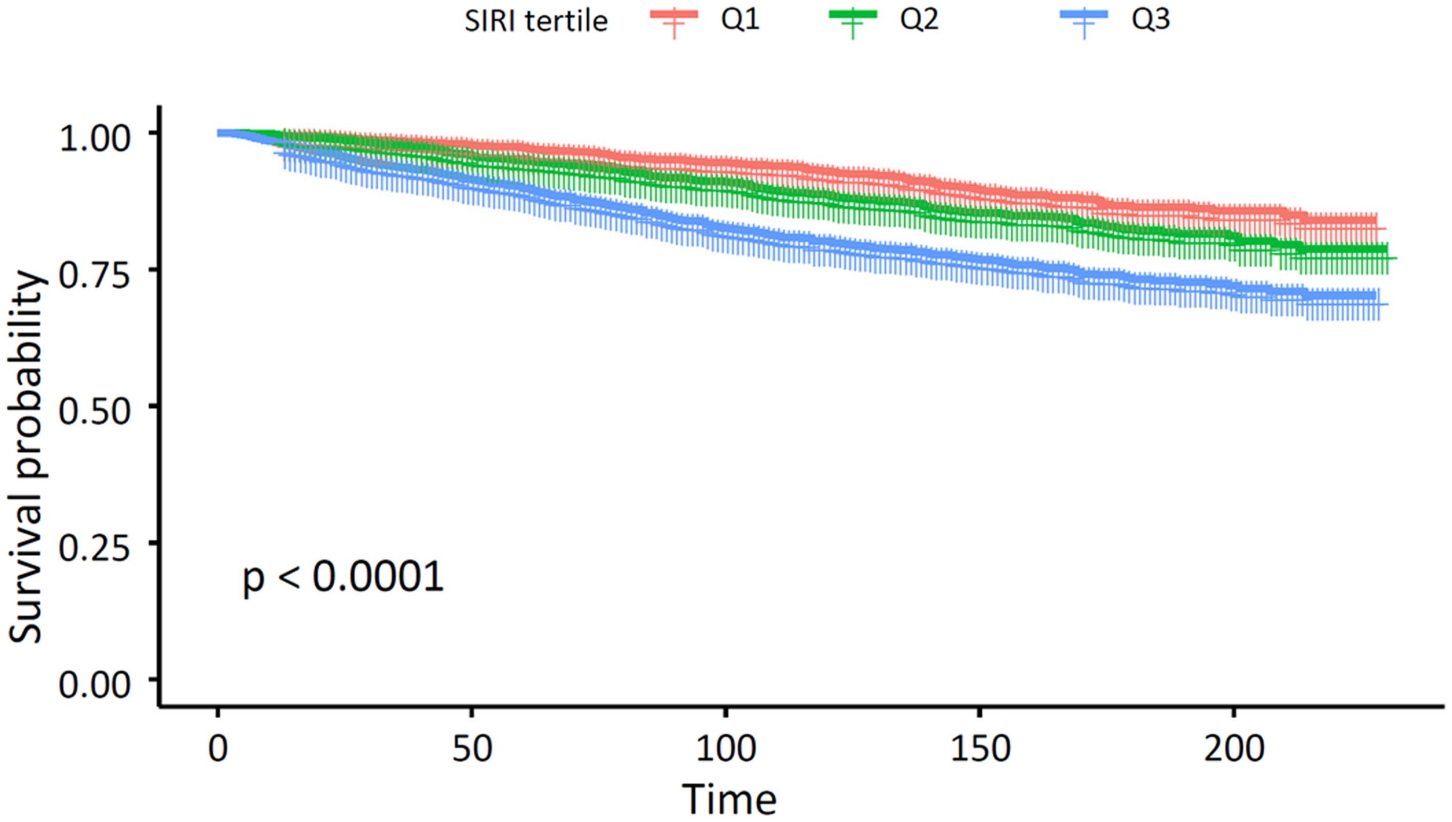
Kaplan–Meier survival analysis plot for all-cause mortality with SIRI categories.

**Figure 4:**
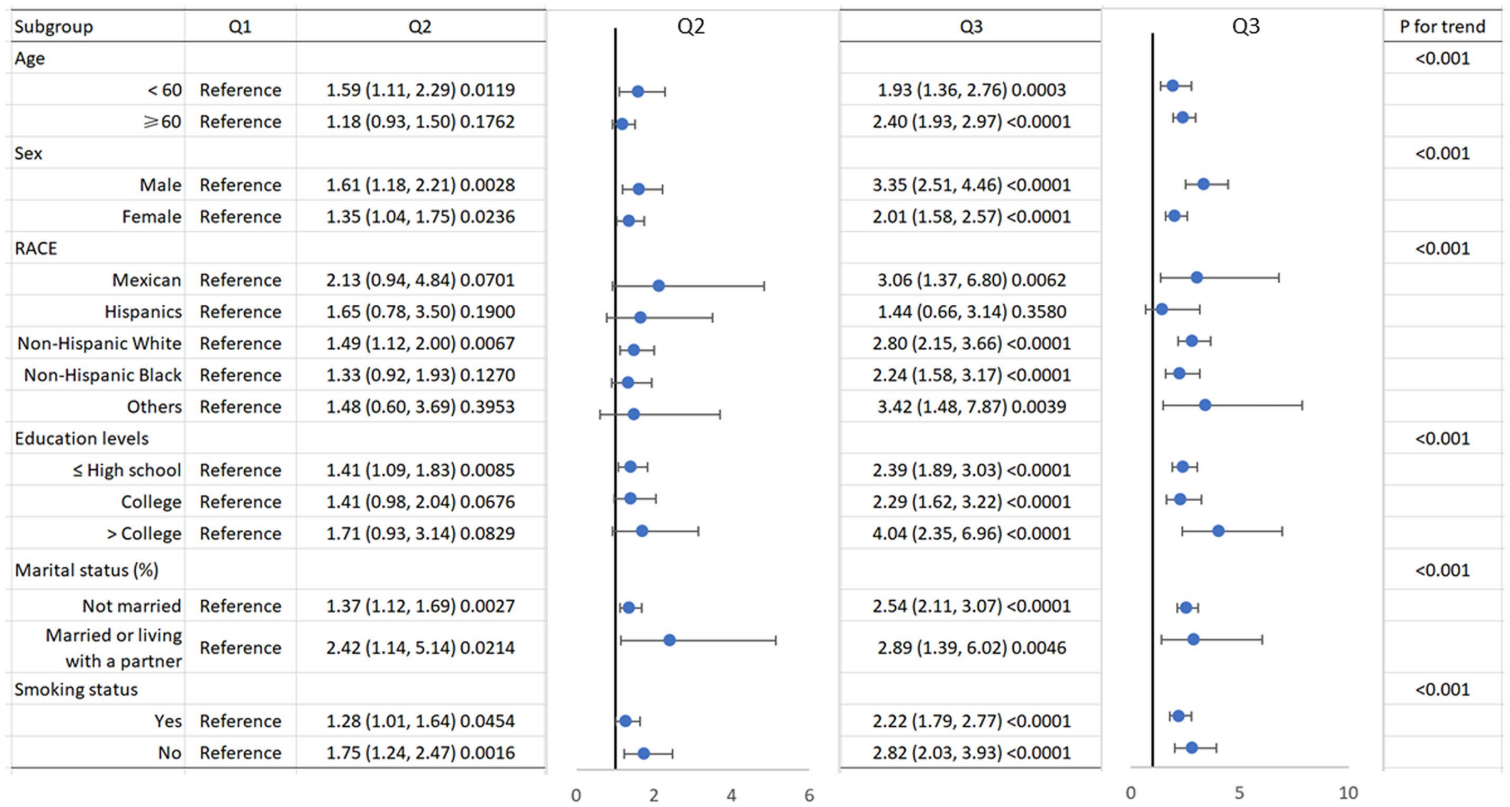
Subgroup of the association between systemic inflammation response index and all-cause mortality.

Table 2 displays the three quartiles of all-cause mortality rates in asthma patients at different levels of SIRI. In the unadjusted Model 1, the hazard ratios (HRs) from the lowest SIRI tertile group to the highest SIRI tertile group were 1.00 (reference), 1.46 (1.20, 1.78), and 2.59 (2.16, 3.11), with a *p*-value of <0.001, indicating a significant association between SIRI levels and increased overall mortality risk in asthma patients. In Model 2, after further adjusting for age, sex, and race, the HRs for the SIRI tertile groups were 1.00 (reference), 1.26 (1.03, 1.54), and 2.19 (1.81, 2.65), with a *p*-value of <0.001. The risk of overall mortality gradually increased with increasing SIRI. In Model 3, after further adjusting for marital status, education level, and smoking status based on Model 2, the results of Model 3 were similar to those of Model 2. The HRs for the SIRI tertile groups were 1.00 (reference), 1.24 (1.01, 1.52), and 2.08 (1.71, 2.51), with a *p*-value of <0.001. Thus, the relationship between SIRI and overall mortality in asthma patients remained consistent in different models of our study. We then used a smoothing curve to plot the relationship between SIRI levels and mortality risk in asthma patients. We found an approximately linear relationship between SIRI and overall mortality risk, and the risk of overall mortality increased with an increase in SIRI (Figure 2). Furthermore, we conducted a Kaplan–Meier survival analysis for the overall mortality rates among the SIRI tertile groups. The curves showed that, compared to the higher SIRI tertile groups, the lowest SIRI tertile group was associated with the lowest lifetime risk of overall mortality. As the SIRI tertiles increased, the risk of overall mortality also increased (Figure 3). Finally, we performed a subgroup analysis on the association between SIRI and asthma and found that higher SIRI levels were significantly associated with an increased risk of all-cause mortality, regardless of age (<60 or ≥60), sex (male or female), race, education level, smoking status, and marital status (*p* < 0.001) (Figure 4).

**Table 2:** Multivariate Cox regression analysis of systemic inflammation response index with all-cause mortality.

SIRI	Model 1 HR (95% CI) *P*-value	Model 2 HR (95% CI) *P*-value	Model 3 HR (95% CI) *P*-value
Q1	Reference	Reference	Reference
Q2	1.46 (1.20, 1.78) 0.0002	1.26 (1.03, 1.54) 0.0261	1.24 (1.01, 1.52) 0.0378
Q3	2.59 (2.16, 3.11) <0.0001	2.19 (1.81, 2.65) <0.0001	2.08 (1.71, 2.51) <0.0001
*P* for trend	<0.001	<0.001	<0.001

SIRI, systemic inflammation response index; HR, hazard ratio; CI, confidence interval. Model 1 adjusted for none. Model 2 adjusted for age, sex, and race. Model 3 adjusted for age, sex, race, marital status, education level, and smoking status.

## 4 Discussion

By analyzing the NHANES data from 2001 to 2018, we discovered a significant linear correlation between SIRI levels and all-cause mortality rates in patients with asthma. Even after considering potential confounding factors, our analysis results remained robust, indicating that SIRI levels may be independently associated with all-cause mortality rates in asthma patients. This finding introduces an innovative predictive indicator for predicting mortality in asthma patients, offering a fresh perspective for a more comprehensive assessment of their survival risk.

In recent years, inflammatory biomarkers derived from complete blood cell counts, such as neutrophil-to-lymphocyte ratio (NLR), monocyte-to-lymphocyte ratio (MLR), platelet-to-lymphocyte ratio (PLR), systemic immune-inflammation index (SII), and SIRI, have increasingly garnered attention as predictors of asthma. These indices are all based on parameters related to neutrophils, lymphocytes, monocytes, and platelets (12). Zhu et al. found that elevated NLR levels were associated with higher all-cause mortality in asthma in a cross-sectional study from the NHANES database (14). Shi et al. confirmed a correlation between NLR levels and the severity and prognosis of adult asthma and childhood asthma (15, 16). Xuanqi et al. proved that PLR can predict the frequency of acute exacerbations in patients during stable periods with chronic obstructive pulmonary disease (COPD) (17). Additionally, NLR and PLR levels have also been shown to be predictive indices of mortality rates in COPD (18). Moreover, a population-based study has shown that the higher the SII, the poorer the prognosis for middle-aged and elderly patients with COPD and asthma (19). There is evidence to suggest that elevated and activated neutrophil levels are linked to symptom relief and treatment outcomes in symptomatic asthma patients (20), and to the activation of monocytes in the peripheral blood in asthma patients (21). On the one hand, these monocytes can change into macrophages or dendritic cells to promote the inflammatory process and destroy lung function (22–24). On the other hand, lymphocytes, which play a regulatory role in allergic inflammation associated with asthma, may decrease their response due to the increase in neutrophil and monocyte counts (25). According to these findings, utilizing the SIRI to predict the mortality rate of asthma patients is suitable.

Other complete blood count (CBC) parameters, such as eosinophil count and red cell distribution width (RDW), have been suggested to play a significant role in asthma pathobiology. The study by Wenzel et al. indicated that eosinophils are critical in the inflammatory processes of asthma, particularly in patients with severe, corticosteroid-resistant asthma (26). Additionally, other CBC parameters such as RDW have been suggested as markers of hypoxia in COPD. According to a study by Karampitsakos et al., increased RDW is a negative prognostic marker in COPD patients (27). Preliminary findings have also shown promising data regarding the role of RDW in asthma (28). These findings suggest that RDW could potentially be used as a marker of hypoxia and systemic inflammation in asthma, providing a broader perspective on the utility of CBC parameters in managing and understanding asthma.

Asthma and COPD are distinct respiratory conditions with overlapping symptoms, making the differentiation between these diseases and the asthma-COPD overlap syndrome (ACOS) challenging. ACOS represents a condition where patients exhibit clinical features of both asthma and COPD, often leading to more severe symptoms and complications. The diagnosis and management of ACOS are complex due to the lack of standardized criteria and biomarkers. Karampitsakos et al. proposed fractional exhaled nitric oxide (FeNO) as a potential biomarker to aid in distinguishing these conditions (29). Thus, identifying clinically applicable biomarkers of inflammation in asthma, specifically mentioning the role of FeNO and other potential biomarkers such as eosinophil count and RDW, is important for further research in these areas.

The strengths of this study are as follows: First, our study is the first to investigate the association between SIRI levels and mortality risk in patients with asthma. Second, our research included 6,156 participants, providing a large dataset for statistical analysis. Third, we also covered potential risk factors, which make our statistical results more credible. However, there are limitations to consider in our study. The NHANES database is based on cross-sectional survey data; this type of survey data focuses on specific time points but does not establish causality or track the long-term relationship between asthma and mortality. Accordingly, future studies using longitudinal data will provide stronger evidence for this evaluation. Although we had adjusted for several potential confounding factors, there were still some unmeasured confounders we missed. The biological mechanisms that potentially link SIRI to increased mortality in asthma remain unclear. Therefore, future research aimed at exploring these mechanisms is necessary.

In summary, our study provides evidence that higher SIRI levels are relevant to the increased all-cause mortality in patients with asthma. However, further research is warranted to confirm our findings.

## Data Availability

The datasets presented in this study can be found in online repositories. The names of the repository/repositories and accession number(s) can be found below: Publicly available datasets were analyzed in this study. This data can be found at: https://www.cdc.gov/nchs/nhanes/index.htm.
